# Neonatal overfeeding alters adult anxiety and stress responsiveness

**DOI:** 10.1016/j.psyneuen.2009.02.013

**Published:** 2009-09

**Authors:** Sarah J. Spencer, Alan Tilbrook

**Affiliations:** Department of Physiology, Faculty of Medicine, Monash University, Melbourne, Victoria 3800, Australia

**Keywords:** Anxiety, HPA axis, Obesity, Sex, Stress

## Abstract

The neonatal nutritional environment is critical for programming the systems regulating body weight, and an inappropriate nutritional environment in early life can lead to overweight or obesity later on. In this study we demonstrate that changes to the neonatal nutritional environment, induced by changing the litter size in which the animal is raised, can alter not only body weight into adulthood, but also anxiety behaviours and stress responsiveness. These effects appear to be somewhat sex-dependent, affecting female rats more strongly than males. Thus, Wistar rats raised in small litters, where they have greater access to their mothers’ milk, weigh more in adulthood than those raised in large litters. Females from these small litters show enhanced exploratory behaviour and reduced anxiety in the elevated plus maze, entering the open arms more often. They also display enhanced central responses to restraint stress including greater activation of the paraventricular nucleus of the hypothalamus and paraventricular nucleus of the thalamus, possibly indicating that the changes we see are related to enhanced arousal. Thus, while neonatal litter size affects long-term body weight regulation, it can also alter long-term activity, anxiety and stress responsiveness, and the degree to which it does so appears to be sex-dependent.

## 1 Introduction

Nutrition during the perinatal period is crucial in programming the regulatory systems underlying the long-term maintenance of body weight. Significant alterations to the perinatal nutritional environment can lead to a predisposition to obesity as well as a variety of related diseases and disorders throughout later life. In humans, maternal over- and under-nutrition during gestation can predispose the offspring to become overweight adults (Ravelli et al., 1976, 1999; Kensara et al., 2005; Muhlhausler et al., 2006; Taylor and Poston, 2007; Wrotniak et al., 2008), leading to an increased likelihood of type two diabetes mellitus, cardiovascular disease, cancers, and other diseases (Flegal et al., 2007; Crowley, 2008). Animal models, such as intrauterine growth restriction and pre- and post-natal dietary manipulations, have yielded similar findings, with both maternal over- and under-nutrition during gestation resulting in offspring with overweight phenotypes (e.g. Taylor and Poston, 2007). The suckling period is equally crucial. Maternal over-nutrition during lactation can program post-weaning obesity in rat pups, as can neonatal overfeeding caused by reducing litter sizes and therefore competition for food (Schmidt et al., 2001; Morris et al., 2005; Rodel et al., 2008).

In addition to co-morbidities such as type two diabetes, overweight animals, including humans, are also more likely to develop affective disorders like anxiety or depression (Doyle et al., 2007; Scott et al., 2008). For instance, a New Zealand study in humans determined a significant association between obesity and mood disorders with a particularly strong association with anxiety (Scott et al., 2008). Furthermore, overweight individuals can manifest dysregulation of their “stress” hypothalamic–pituitary–adrenal (HPA) axes (Rosmond et al., 1998; Jessop et al., 2001; Boullu-Ciocca et al., 2005). As such, male rats raised in a hyper-nutritive environment, such as in a small litter, demonstrate accelerated HPA axis maturation, accompanied by elevated stress-induced corticosterone secretion in adulthood (Boullu-Ciocca et al., 2005). Conversely, there is also evidence to suggest the involvement of elevated glucocorticoids in the development and maintenance of obesity (Adam and Epel, 2007; Henry and Clarke, 2008), in a potentially self-perpetuating cycle.

It is interesting to note that this predisposition to developing affective disorders and HPA axis dysregulation in the overweight and obese may be sex-dependent. As well as males and females in general displaying different intensities of glucocorticoid response to most stressors (Turner et al., 2002; Kudielka and Kirschbaum, 2005; Roca et al., 2005), obesity in humans can lead to an up-regulation of HPA axis responses to stress, particularly in men (Vicennati et al., 2006; Pasquali et al., 2008). Females also have a greater up-regulation of 11β-hydroxysteroid dehydrogenase-1 in obesity than do males (Paulsen et al., 2007), leading to relatively increased activity of glucocorticoids in adipose tissue. These changes are reflected in greater alterations in glucocorticoid negative feedback in obese females than obese males (Pasquali et al., 2002). These sex-dependent changes that occur with increased adiposity may be linked to differences in the incidence of depression and anxiety that have been reported between obese males and females (Rosmond and Bjorntorp, 1998; Mahony, 2008). Unfortunately, most studies into overweight/obesity have been conducted using male animal models and so little is known about how the HPA axis changes in female rodents subjected to different nutritional environments.

In the present investigation we therefore hypothesized that alterations to the neonatal nutritional environment, as induced by raising the rats in litters of different sizes, would alter behavioural manifestations of anxiety in adult rats and this would be reflected in changes to central regulation of HPA axis function, stress responsiveness and anxiety as determined by glucocorticoid production and brain Fos-immunoreactivity. We further hypothesized that the overfeeding due to different litter sizes would have differential impacts on male and female rats. To explore this question we employed a litter-size manipulation model to alter the neonatal nutritional environment to which the rats were exposed (Schmidt et al., 2001; Morris et al., 2005; Rodel et al., 2008). Thus, male and female rats from small litters (SL; postnatal overfeeding) were compared with those from large litters (LL) in tests for activity, anxiety and HPA axis and central responsiveness to stress.

## 2 Materials and methods

### 2.1 Animals

Timed pregnant Wistar rats were obtained from the Animal Resources Centre, WA, Australia. They were maintained at 22 °C on a 12 h light/dark cycle (07:00–19:00 h) with pelleted rat chow and water available *ad libitum*. All procedures were conducted in accordance with the National Health and Medical Research Council Australia Code of Practice for the Care of Experimental Animals and were approved by the Monash University School of Biomedical Sciences Animal Ethics Committee.

### 2.2 Litter manipulation

On the day of birth (postnatal day (P) 1) all pups were removed from their dams and randomly reallocated to new dams in litters of four or 16. Care was taken that no dam received any of her own pups and each new litter was made up of 50% males and 50% females. Excess pups were culled, as were any litters (*n* = 1) where eight or fewer or 16 or more pups were born.

Following pup reallocation, the litters were weighed weekly as whole litter units, it having previously been determined that males and females show similar growth rates until after weaning (Spencer et al., 2007), at which time the pups were separated into same-sex pairs. The rats were otherwise left undisturbed, except for the usual cleaning and feeding procedures and weekly weighing, until experimentation. In these experiments we used 106 offspring from 26 litters. Every experimental group contained representatives from at least two litters.

### 2.3 Food intake

From P60 to P67 the males (13 pairs of offspring from 5 SL and 2 LL) and P63 to P70 the females (13 pairs of offspring from 5 SL and 2 LL) were assessed for basal food intake and weight gain in a one-week period.

### 2.4 Elevated plus maze test for activity and anxiety

At P60 a subset of the males (29 offspring from 6 SL and 3 LL) and P63 the females (27 offspring from 6 SL and 3 LL) was tested for 7 min in the elevated plus maze test for activity and anxiety in a novel environment, as described previously (Spencer et al., 2005b). The plus maze was made of wood, painted black and was raised 50 cm above the floor. It consisted of two opposite open arms of 50 cm × 15 cm and two closed arms of the same dimensions with 15 cm high walls. Each rat was placed in the centre of the plus maze and filmed and later scored by an experimenter blinded to the rats’ treatments for the number of entries into and percentage time spent in each of the open and closed arms, and the incidences of vertical exploration (rearing). The rat was regarded as having moved into an arm when all four paws had crossed the threshold of the arm. The maze was thoroughly cleaned with 70% ethanol between trials.

### 2.5 Open field test for locomotor activity and anxiety

At P60 or P61 a separate group of animals (16 males from 3 SL and 4 LL and 22 females from 6 SL and 2 LL) was tested for locomotor activity and anxiety using a mini open field paradigm as described previously (Spencer et al., 2005b). The circular open field was of 50 cm diameter and had dark green inside walls 90 cm high. The arena was placed on a cream-coloured floor with a black grid that divided the area into four sections. Each animal was placed in the centre of the arena and filmed and later scored, by an experimenter blinded to the rats’ treatment, for locomotion (number of grid-lines crossed), number of entries into the middle of the arena, instances of vertical exploration (rearing), and number of instances of and time spent grooming, in a period of 10 min. The open field was thoroughly cleaned with 70% ethanol between trials.

### 2.6 Femoral artery catheterization surgery

At approximately P70 a subset of the rats was implanted with a catheter in the left femoral artery for blood sampling as has been previously described (Spencer et al., 2005a). Briefly, rats were anaesthetized with isofluorane, induced at 5% and maintained at 2%, and a silastic-tipped vinyl catheter inserted into the artery, routed under the skin and externalized at the back of the neck. Catheters were filled with heparinized saline (100 units/ml) containing gentamicin (20 mg/ml; Sigma–Aldrich, Castle Hill, NSW, Australia). The rats were housed singly after surgery and were left to recover undisturbed for at least four days before experimentation. Catheters were flushed daily with heparinized saline to ensure patency.

### 2.7 Stressor setup, application and blood sampling

On the day of the stressor experiment, the rats (15 males from 5 SL and 3 LL and 18 females from 6 SL and 2 LL) were brought into the testing room at 07:00 h and allowed 2 h to acclimatize to the room. A baseline blood sample was taken immediately prior to the 15 min restraint stress (restraint of the rat in a ventilated Perspex tube, 7 cm in diameter, 24 cm in length, with an adjustable restraining length between 10 and 18 cm). At the end of the restraint period, catheters were attached to a syringe outside the cage to allow blood sampling without further handling. Blood samples (0.4 ml, immediately replaced with an equal volume of heparinized saline) were collected immediately prior to and 30 and 60 min after onset of the stressor. Blood samples were kept on ice until the end of the experiment, when they were centrifuged and the plasma aliquots stored at −20 °C until assayed.

### 2.8 Tissue processing

Two hours after the onset of the stressor, or equivalent, rats (24 males from 6 SL and 3 LL, 4 non-stressed and 8 stressed for each group, and 20 females from 5 SL and 3 LL, 4 non-stressed and 8 stressed for each) were deeply anaesthetized with Lethabarb (approximately 150 mg/kg pentobarbitone sodium, intraperitoneal) and perfused transcardially with phosphate buffered saline (PBS; 4 °C, pH 7.4) followed by 4% paraformaldehyde in PBS (4 °C, pH 7.4). Brains were then removed and post-fixed for 24 h in the same fixative before being cryoprotected with 20% sucrose in PBS (4 °C). Forebrains were subsequently cut using a cryostat into 40 μm coronal sections.

Neuronal activation was assessed on the basis of positive Fos-immunoreactivity, seen as a black deposit in the nucleus. Briefly, a one-in-four series of forebrain sections from each animal was incubated in primary Fos antibody (48 h; 1:10,000; rabbit polyclonal; Santa Cruz Biotechnology, Santa Cruz, CA), then in secondary antibody (2 h; 1:200; biotinylated anti-rabbit; Vector Laboratories, Burlingame CA) and in an avidin–biotin horseradish peroxidase (HRP) complex (1 h; Vector Elite kit, Vector). The sections were then incubated in nickel diaminobenzidine to visualize the HRP activity, seen as a black nuclear deposit. The reaction was terminated once an optimal contrast between specific cellular and non-specific background labelling was reached. Sections from each treatment group were processed simultaneously. Sections were mounted on polylysine-coated slides, dehydrated in a series of alcohols, cleared in Histoclear and coverslipped.

### 2.9 Corticosterone assay

A standard corticosterone enzyme immunoassay kit (Diagnostic Systems Laboratories, Inc., Texas, USA), was used to assess plasma corticosterone. The inter-assay variability for this assay was 3.7–6.1% coefficient of variation (CV), intra-assay variability 2.3–3.9% CV, and lower limit of detection 1.6 ng/ml. Samples from all treatment groups were assayed together in duplicate.

### 2.10 Data analysis

Pre-weaning body weights between LL and SL rats were compared using a one-way analysis of variance (ANOVA) with repeated measures, with litter size as the between factor and time as the repeated measure. When a significant interaction was found between litter size and time, Student's unpaired *t*-tests were performed for each time point. Comparisons between males and females and LL and SL adult body weights, food intake, and each parameter of the elevated plus maze and open field were performed using two-way ANOVAs with sex and litter size as between factors. When a significant interaction was found between sex and litter size, Tukey's HSD *post hoc* tests were performed comparing each group. Adult corticosterone levels were compared in the same way but included time as a repeated measure. Counts of Fos-immunoreactive cells were compared using multi-factor ANOVAs for each brain region, with sex, litter size and stress as between factors. An experimenter, blinded to the group treatments, carried out counts of cells positive for Fos-immunoreactivity in regions of interest. Numbers of Fos-immunoreactive cells in the paraventricular nucleus of the hypothalamus (PVN) were counted over two sections (∼1.8 and 1.96 mm caudal to bregma), in the medial amygdala (MeA) over four sections (∼2.3–2.94 mm caudal to bregma), in the paraventricular nucleus of the thalamus (PVT) over eight sections (∼1.3–2.58 mm caudal to bregma), and in the lateral hypothalamus (LH) over four sections (∼1.8–2.56 mm caudal to bregma) 160 μm apart.

Photomicrographs were taken using a Zeiss Imager.Z1 microscope and AxioCam HRc digital camera with AxioCam image capture software v4.5, and were cropped and adjusted for intensity and contrast in Corel Photo-Paint. Any intensity and contrast enhancements were performed identically on all photomicrographs.

Data were assessed for homogeneity of variance and transformations applied where appropriate. Data are presented as the mean ± standard error of the mean (SEM). Statistical significance was assumed when *P* < 0.05.

## 3 Results

### 3.1 Body weights

**Figure 1:**
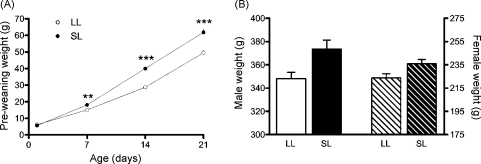
Body weights of rats raised in large (LL) and small (SL) litters. (A) Pre-weaning weights; rats were weighed in whole litter units and weights corrected for the number of pups in the litter; *n* = 4–9 litters/group; **P < 0.01, ***P < 0.001. (B) Postnatal day (P) 63 males and P63 females; *n* = 10–23 rats/group; significant effect of sex *P* < 0.001, significant effect of litter size *P* < 0.001, but no significant interaction. Data are mean + SEM.

As has been previously observed with similar variations on this model (Schmidt et al., 2001; Morris et al., 2005; Rodel et al., 2008), SL rat pups, those raised in a postnatal environment of over-nutrition, weighed significantly more than LL rat pups (Fig. 1A). Pup weights were not different on P1, the day of litter size manipulation, but by P7 the effects of the different litter sizes were evident. Thus, there was a significant interaction between litter size and day (*F*_(3,15)_ = 18.0, *P* < 0.001) and further analysis revealed that SL (*n* = 9 litters per group) were significantly bigger than LL (*n* = 4 litters per group) on days 7 (*P* = 0.002), 14 (*P* < 0.001), and 21 (*P* < 0.001).

The effects on body weight of being raised in a small litter continued to be evident into adulthood (Fig. 1B), and a significant effect of litter size was seen at P63 (*F*_(1,60)_ = 23.1, *P* < 0.001), with SL rats weighing more than their LL counterparts. As expected there was also a significant effect of sex on body weight (*F*_(1,60)_ = 499.8, *P* < 0.001), males being heavier than females, although there was no significant interaction between sex and litter size (*F*_(1,60)_ = 0.04, *P* = 0.9; male LL *n* = 23, SL *n* = 10; female LL *n* = 18, SL *n* = 13).

### 3.2 Food intake

**Figure 2:**
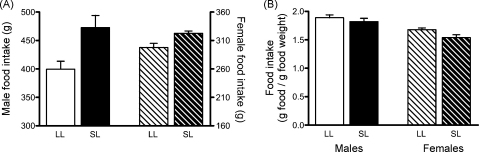
Adult weekly food intake of rats raised in large (LL) and small (SL) litters. (A) Absolute food intake; significant effect of sex *P* < 0.001, significant effect of litter size *P* = 0.001, but no significant interaction. (B) Food intake corrected for body weight; significant effect of sex *P* < 0.001, but no significant interaction. *n* = 5–8 rats/group. Data are mean + SEM.*P < 0.05.

Adult SL rats consumed significantly more rat chow in a one-week period than their LL counterparts (Fig. 2A; *F*_(1,22)_ = 14.0, *P* = 0.001; LL *n* = 8 pairs of males and females, SL *n* = 5 pairs of males and females). There was also a significant sex difference (*F*_(1,22)_ = 91.5, *P* < 0.001) and a trend towards a significant interaction (*F*_(1,22)_ = 3.4, *P* = 0.08). When these values were corrected for body weight, no significant differences between the litter size groups were seen (Fig. 2B, *F*_(1,22)_ = 3.6, *P* = 0.07), but the sex difference was maintained (*F*_(1,22)_ = 28.7, *P* < 0.001), with males consuming more rat chow per gram body weight than females.

### 3.3 Elevated plus maze test for activity and anxiety

**Figure 3:**
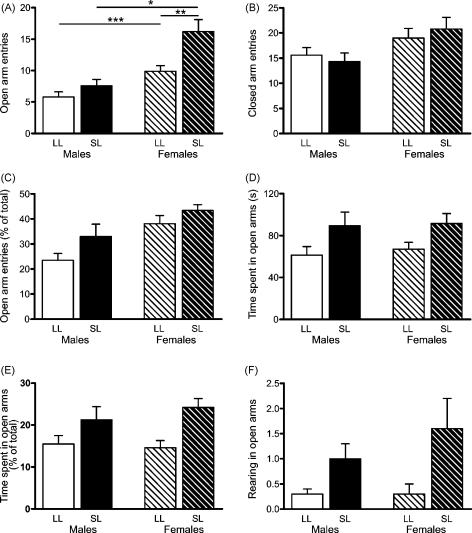
Elevated plus maze performance of rats raised in large (LL) and small (SL) litters. (A) Number of open arm entries; *P < 0.05, **P < 0.01, ***P < 0.001. (B) Number of closed arm entries; significant effect of sex *P* = 0.01, but no significant interaction. (C) Open arm entries as a percentage of total arm entries; significant effect of sex *P* = 0.001, significant effect of litter size *P* = 0.04, but no significant interaction. (D) Time spent in open arms; significant effect of litter size *P* = 0.005, but no significant interaction. (E) Time spent in open arms as a percentage of total time; significant effect of litter size *P* = 0.001, but no significant interaction. (F) Vertical exploration (rearing) in open arms; significant effect of litter size *P* = 0.001, but no significant interaction. Test duration was 7 min. *n* = 11–17 rats/group. Data are mean + SEM.

Behaviour in the elevated plus maze was significantly affected by both sex and the litter size in which the rats were raised (Fig. 3). Thus, female, but not male SL (*n* = 11 and 12 respectively) rats entered the open arms significantly more often than their LL counterparts (*n* = 16 and 17 respectively), there being a significant interaction between sex and litter size (*F*_(1,52)_ = 4.1, *P* = 0.047), and a significant difference between the female (*P* < 0.001; Fig. 3A) but not male (*P* > 0.05) SL and LL groups as determined by *post hoc* tests. Sex differences were also seen in this behaviour, with females being less anxious, *i.e.* doing more exploration of the open arms, than males (male LL vs female LL, *P* = 0.03; male SL vs female SL, *P* < 0.001). Although no interaction between sex and litter size (*F*_(1,52)_ = 0.7, *P* = 0.4) and no effect of litter size (*F*_(1,52)_ = 0.02, *P* = 0.9) was seen on closed arm entries (Fig. 3B), we did see a significant effect of sex (*F*_(1,52)_ = 7.0, *P* = 0.01), determining that females explore the maze more in general. Significant differences between the sexes were also revealed when open arm entries were expressed as a percentage of total entries to account for differences in locomotion (*F*_(1,52)_ = 12.6, *P* = 0.001; Fig. 3C), with female rats making more of their total entries into open arms, again indicating comparatively less anxiety. In this parameter there was no interaction between sex and litter size (*F*_(1,52)_ = 0.4, *P* = 0.5), but there was a significant effect of litter size (*F*_(1,52)_ = 4.4, *P* = 0.04), reflecting a greater percentage of open arm entries in the SL rats. Total time spent in the open arms (*F*_(1,52)_ = 8.7, *P* = 0.005; Fig. 3D), open arm time expressed as a percent of total (*F*_(1,52)_ = 12.1, *P* = 0.001; Fig. 3E), and vertical exploration in the open arms (*F*_(1,52)_ = 11.3, *P* = 0.001; Fig. 3F) were significantly affected by litter size—the SL rats spending more of their time exploring the open arms and doing more vertical exploration than the LL. However, in these parameters, there were no effects of sex and no significant interactions (*P* > 0.3 for each).

### 3.4 Open field test for activity and anxiety

**Figure 4:**
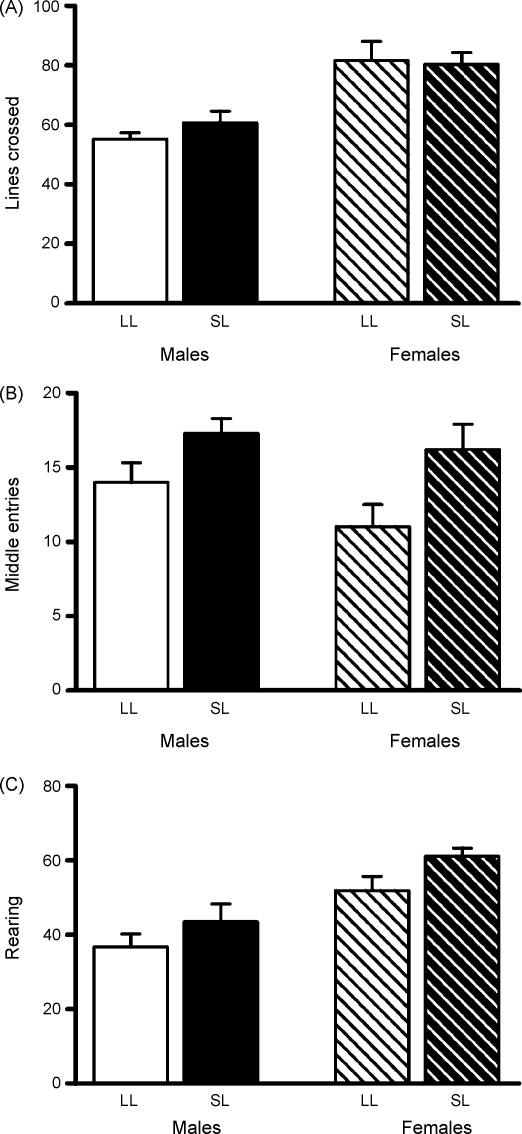
Open field performance of rats raised in large (LL) and small (SL) litters. (A) Locomotion (total number of lines crossed); significant effect of sex *P* < 0.001, but no significant interaction. (B) Middle arena exploration (number of entries into the middle); significant effect of litter size *P* = 0.001, but no significant interaction. (C) Vertical exploration (rearing); significant effect of sex *P* < 0.001, significant effect of litter size *P* = 0.03, but no significant interaction. Test duration was 10 min. *n* = 6–12 rats/group. Data are mean + SEM.

As with the elevated plus maze, differences due to sex and litter size were seen in behaviours in the open field. Rats from SL (males *n* = 6, females *n* = 12) ventured into the middle of the open field significantly more often than those from LL (males and females *n* = 10; *F*_(1,34)_ = 12.2, *P* = 0.001; Fig. 4B), further indicating reduced anxiety in this group. As with the elevated plus maze, SL rats also performed more vertical exploration (*F*_(1,34)_ = 5.2, *P* = 0.03; Fig. 4C). There was no significant interaction between sex and litter size in either of these parameters (*P* > 0.2 for each), but we did see a significant effect of sex on vertical exploration (*F*_(1,34)_ = 22.2, *P* < 0.001) and a significant effect of sex on total exploration of the open field (*F*_(1,34)_ = 23.6, *P* < 0.001; Fig. 4A).

### 3.5 Corticosterone responses to restraint stress

**Figure 5:**
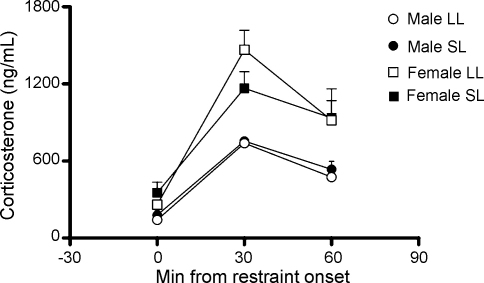
Plasma corticosterone responses to restraint in rats raised in large (LL) and small (SL) litters; significant effect of sex *P* = 0.001, significant effect of stress *P* < 0.001, significant effect of sex × time *P* = 0.02, but no significant three-way interaction. *n* = 5–12 rats/group. Data are mean + SEM.

No significant differences in corticosterone levels were seen between the litter size groups (LL *n* = 5 males and 12 females, SL *n* = 10 males and 6 females; Fig. 5). There was a significant effect of the restraint stress on corticosterone (*F*_(2,58)_ = 57.5, *P* < 0.001), indicating that the stressor did elevate corticosterone as expected. There was also a significant effect of sex (*F*_(1,29)_ = 20.8, *P* = 0.001) and significant interaction between sex and time (*F*_(2,58)_ = 4.1, *P* = 0.02), indicating possible sex differences in the amount of corticosterone produced, but there was no significant three-way interaction (sex × litter size × time, *F*_(2,58)_ = 0.8, *P* = 0.4) or sex × litter effect (*F*_(1,29)_ = 0.4, *P* = 0.5).

### 3.6 Central responses to restraint stress

#### 3.6.1 Paraventricular nucleus of the hypothalamus

**Figure 6:**
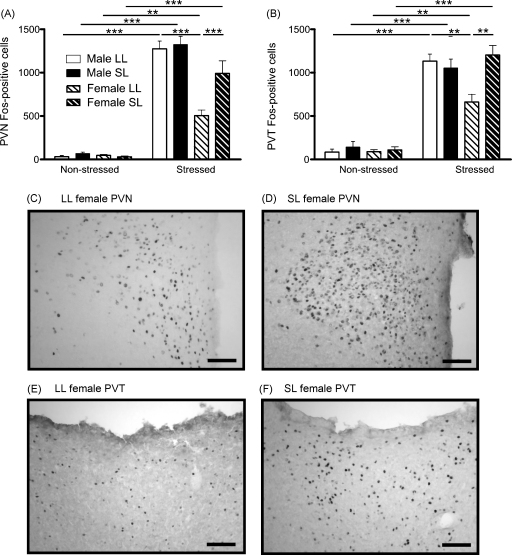
Neuronal activation after restraint in rats raised in large (LL) and small (SL) litters. (A) Neuronal activation in the paraventricular nucleus of the hypothalamus (PVN), as assessed by numbers of Fos-immunoreactive cells; **P < 0.01, ***P < 0.001. (B) Number of Fos-immunoreactive cells in the paraventricular nucleus of the thalamus (PVT); **P < 0.01, ***P < 0.001. (C) Representative photomicrograph of the PVN of an LL female. (D) Representative photomicrograph of the PVN of an SL female. (E) Representative photomicrograph of the PVT of an LL female. (F) Representative photomicrograph of the PVT of an SL female. *n* = 4–8 rats/group. Data are mean + SEM. Scale bars = 100 μm.

Analysis of Fos-immunoreactivity in the PVN revealed a significant effect of being raised in a small litter on female (non-stressed LL and SL *n* = 4; stressed LL and SL *n* = 6; Fig. 6A, C, and D) but not male rats (non-stressed LL and SL *n* = 4; stressed LL and SL *n* = 8). There was a significant interaction between sex, litter size and stress (*F*_(1,36)_ = 6.3, *P* = 0.02). In both groups of male rats the PVN was activated after restraint (*P* < 0.001 each) but the litter size did not affect the magnitude of these responses (*P* > 0.05). In the females, on the other hand, the neonatal litter size did fundamentally affect adult central responsiveness to restraint stress. Both the LL (*P* = 0.007) and SL (*P* < 0.001) groups showed PVN activation after restraint but the number of activated cells was significantly greater in the SL rats (*P* < 0.001). *Post hoc* sex differences were also seen in the PVN, with the male and female LL but not SL groups being significantly different from one another (LL male vs female, *P* < 0.001; SL male vs female, *P* > 0.05).

#### 3.6.2 Medial amygdala

Such differences as were seen between the litter sizes in the PVN after restraint stress in the females were not reflected in similar changes to the psychological stress-processing limbic region, the MeA. Thus, we saw a significant main effect of stress (*F*_(1,36)_ = 70.5, *P* < 0.001) but no significant three way interaction (*F*_(1,36)_ = 0.7, *P* = 0.4) and no significant effect of litter size (*F*_(1,36)_ = 0.9, *P* = 0.4) on Fos between the groups (Males—non-stressed LL: 104 ± 44.1, SL: 183.8 ± 105.2; stressed LL: 691.1 ± 65.2, SL: 660.5 ± 98.2. Females—non-stressed LL: 176 ± 62.2, SL: 220 ± 95.8; stressed LL: 815.4 ± 95.3, SL: 999.4 ± 112.3 Fos-immunoreactive cells).

#### 3.6.3 Paraventricular nucleus of the thalamus

The possibility that the PVN hyper-responsiveness of the female SL rats may be attributed to enhanced arousal or attention led us to examine Fos-immunoreactivity in the PVT. Restraint caused similar responses in the PVT as in the PVN, with a three-way interaction between sex, litter size and stress (*F*_(1,36)_ = 7.3, *P* = 0.01; Fig. 6B, E, and F). Restraint stress enhanced numbers of PVT cells expressing Fos-immunoreactivity in both the LL (*P* = 0.001) and SL (*P* < 0.001) groups for both males and females. In the males there was a significant *post hoc* effect of stress but not litter size on PVT responses to restraint (*P* < 0.001 each stress, *P* > 0.05 each litter size). In the females there was also an effect of having being raised in a small litter, SL rats had significantly more Fos-immunoreactive cells after restraint in the PVT than the LL females (*P* = 0.001). *Post hoc* sex differences were also seen in the PVT, with the male and female LL but not SL groups being significantly different from one another (LL male vs female, *P* = 0.003; SL male vs female, *P* > 0.05).

#### 3.6.4 Lateral hypothalamus

The PVN-projecting LH was also examined and although there was a significant effect of the restraint stress (*F*_(1,36)_ = 107.6, *P* < 0.001), there was no significant three way interaction (*F*_(1,36)_ = 1.4, *P* = 0.3) and no differences between the LL and SL groups (*F*_(1,36)_ = 0.009, *P* > 0.9; Male—non-stressed LL: 41.8 ± 23, SL: 78.5 ± 22.5; stressed LL: 713.8 ± 50.3, SL: 603 ± 61.4. Female—non-stressed LL: 61.8 ± 14.8, SL: 41.3 ± 8.5; stressed LL: 666.9 ± 106.6, SL: 785.2 ± 143.3 Fos-immunoreactive cells).

## 4 Discussion

The neonatal nutritional environment, as induced by altering the litter size in which the animals were raised, can clearly have important implications for long-term physiology. In the current study we have demonstrated that changes to the neonatal nutritional environment can alter not only body weight into adulthood, but also anxiety behaviours and stress responsiveness. Interestingly, these effects appear to be sex-dependent, in many aspects affecting female rats more strongly than males.

Human studies have established that obesity is linked to an hyperactive HPA axis (Vicennati et al., 2006), with subjects displaying elevated basal and stress-induced cortisol and adrenocorticotropic hormone (ACTH). In the present investigation, we saw no effect of neonatal litter size on basal or stress-induced corticosterone concentrations. However, we did see increased activation, as determined by Fos-immunoreactivity, of the PVN in females that were raised in small litters and therefore manifested an overweight phenotype in adulthood. A good correlation between numbers of PVN cells activated (*i.e.* Fos-immunoreactive) by restraint stress and numbers of PVN corticotropin-releasing hormone (CRH) cells activated (*i.e.* Fos- and CRH-immunoreactive) by restraint stress has previously been reported (Dayas et al., 1999), leading us to regard PVN Fos-immunoreactivity as representative of activation of the apex of the HPA axis. It was unexpected, although not unprecedented, that this enhanced PVN response to stress was not accompanied by a corresponding enhanced corticosterone response. We have previously reported a similar dissociation between the neural and downstream components of the HPA axis (Spencer et al., 2005a). Potential explanations for this dissociation include changes to CRH/arginine vasopressin signalling and interactions, pro-opiomelanocortin and ACTH production, adrenal sensitivity, and glucocorticoid metabolism (e.g. Tilbrook and Clarke, 2006), all of which could potentially be affected by the neonatal environment in our rats.

Unlike in the work of Boullu-Ciocca et al. (2005), we did not see differences in corticosterone after stress. Possible differences in experimental design, such as with the stressor applied, in their case shaking platform, could be responsible for this. Alternatively, the sex composition of the litter has been shown to affect adult physiology in rats (Michaels and Holtzman, 2006). Boullu-Ciocca's experiments were conducted using rats raised in single sex (male only) litters (Boullu-Ciocca et al., 2005), potentially yielding different results.

In the present investigation, we also saw long-term effects of neonatal overfeeding due to a small litter environment on behaviours in tests of anxiety. Anxiety and adiposity are intimately associated in humans, with even the moderately overweight being more likely to exhibit anxiety (Barry et al., 2008; Petry et al., 2008). This association is particularly reported in females (Mahony, 2008). Obese women are more likely to report a history of anxiety than are obese men, and have higher scores in the PsyBari Social Anxiety Index (Mahony, 2008). These findings in humans led us to speculate that this may also be true in a rodent model. However, contrary to our expectations, female rats raised in small litters, where they had greater access to their mother's milk and subsequently displayed greater body weight gain, did not show indications of being more anxious than their lean counterparts. Indeed, these small-litter rats showed indications of reduced anxiety compared with large litters as demonstrated by enhanced open arm exploration in the elevated plus maze, and greater vertical and middle arena exploration and reduced grooming in the open field.

It should be noted that the relationship between weight gain and affective disorders in humans is complex and it is unclear whether anxiety is primarily an effect of overweight/obesity in these cases, or a contributing cause (Nieuwenhuizen and Rutters, 2008). Patients with pre-existing anxiety disorders have been found to exhibit smaller decreases in body mass indices following gastric bypass surgery than those without such disorders (Kalarchian et al., 2008), possibly indicating that anxiety contributes to at least the maintenance of obesity. It is also possible that the social implications of being obese contribute as much to anxiety in this population as the obesity itself (Young-Hyman et al., 2006; Jansen et al., 2008), a factor that is unlikely to play a role in rodent models.

Other models of overweight and obesity in rodents have, however, also demonstrated increased levels of anxiety with increased body weight. For example, Souza et al. (2007) showed that rats become overweight when given highly palatable food, and that this is associated with increases in some anxiety-like behaviours. Agouti protein over-expression leads to obesity in mice, and this too is associated with anxiety (Harris et al., 2001). On the other hand, obese Zucker rats do not exhibit anxiety-like behaviours when compared with lean Zucker rats (Chaouloff, 1994), and rats bred specifically for anxious traits do not always differ from their low anxiety counterparts in adult body weight (Bosch et al., 2006). Thus, the relationship between affect and weight is complex and not readily explained by existing literature.

A partial explanation for the reduced anxiety-like behaviours we see in our rats raised in small litters may be the degree of maternal attention they received during the neonatal period. In addition to having reduced competition for food, the rats raised in litters of only four animals would also have had reduced competition for maternal attention. It is known that maternal attention can have pronounced long-term effects on the animal, with the offspring of mothers who impart more intense nursing and grooming developing to have reduced behavioural manifestations of fear and reduced HPA axis responses to stress (Liu et al., 1997; Caldji et al., 1998; Francis et al., 1999; Francis and Meaney, 1999; Fish et al., 2004). It has been shown that the amount of high intensity nursing and grooming a rat displays is not dependent upon litter size, suggesting that the dams spend approximately the same time in such behaviours irrespective of the number of pups, thereby imparting more of her attention on each pup in the smaller litters (Champagne et al., 2003). Interestingly, rats raised by high intensity nursing mothers display enhanced exploration of novel environments (Caldji et al., 1998), similar to our small litter females. It is possible, therefore, that maternal attention contributes to our results via attention-induced alterations in rats raised in small litters. An important confounding factor in this argument, however, is that we did not see an attenuation of HPA axis responses to stress in these animals. Previous studies examining effects of maternal care show attenuated HPA axis responses in adult offspring exposed to high levels of maternal care during development (Liu et al., 1997; Francis et al., 1999; Francis and Meaney, 1999; Fish et al., 2004). Our female rats by contrast were more reactive in their PVN response to restraint stress. We would also expect to see the changes to the same degree in the male rats if maternal care was a major contributing factor here.

In addition to its role in the HPA axis, the PVN is also a crucial region involved in arousal, wakefulness and attentional processes (Smith et al., 2006; Szentirmai et al., 2007; Kita et al., 2008). It receives projections from, among other regions, the PVT (Ferguson et al., 1984), an area that is thought to be important in translating homeostatic and viscerosensory information into arousal, attention, and mood. As we have seen, the activation of the PVT in our small litter females was significantly enhanced after restraint stress and this cannot be explained by a generalized enhanced increase in neuronal activation as we did not see differences in the MeA, a region usually associated with processing responses to psychological stress (Dayas et al., 1999, 2001), or the LH. It is therefore feasible that the enhanced PVN Fos and elevated plus maze open arm exploratory behaviour that we see in our female rats from small litters could be due to enhanced levels of arousal or wakefulness in these animals. Interestingly, there is some precedent for sex-specific changes in this system. For example, the orexin/hypocretin neurons in the LH (Kirouac et al., 2005; Huang et al., 2006; Pasumarthi and Fadel, 2008) are involved in enhancing activity and arousal, as well as having a role in activation of the PVN associated with arousal (Sato-Suzuki et al., 2002; Brunton and Russell, 2003; Kotz, 2006), and this system has been shown to be sexually dimorphic, with females having higher levels of prepro-orexin and orexin receptor mRNA in their hypothalami than males (Johren et al., 2001, 2002). Similarly, ghrelin, also implicated in attention and arousal (Szentirmai et al., 2007), can be altered in a sexually dimorphic manner by a high fat diet (Priego et al., 2008). Although we did not see differences in absolute numbers of activated LH neurons, our present findings of enhanced exploration in tests of anxiety in conjunction with enhanced PVN responses to stress could potentially be explained by alterations in the orexin and/or ghrelin-based arousal systems. An up-regulation of such signalling to the PVT/PVN in our small litter females could potentially lead to enhanced exploratory activity in the elevated plus maze and open field in addition to an hyperactive PVN response to stress.

In this investigation we have therefore demonstrated that the neonatal environment can play a crucial role in programming the adult phenotype as well as behavioural and central responses to novel or stressful stimuli, an affect particularly pronounced in females. We suggest that these changes may be due, in part, to alterations in arousal and attention processed via the PVT.

## Role of funding sources

This work was supported by the National Health and Medical Research Council (NHMRC) of Australia and the Wellcome Trust (UK). S.J.S. holds a Peter Doherty Fellowship awarded by the NHMRC of Australia (465167). These funding agencies had no further role in study design, in the collection, analysis and interpretation of data, in the writing of the report or in the decision to submit the paper for publication.

## Conflicts of interest

None declared.
